# Supplementary figures and images for: TeXP: Deconvolving the effects of pervasive and autonomous transcription of transposable elements
Source: PLoS Comput Biol. 2019 Aug 19;15(8):e1007293. doi: 10.1371/journal.pcbi.1007293 (PMC6715295; doi:10.1371/journal.pcbi.1007293)

A.

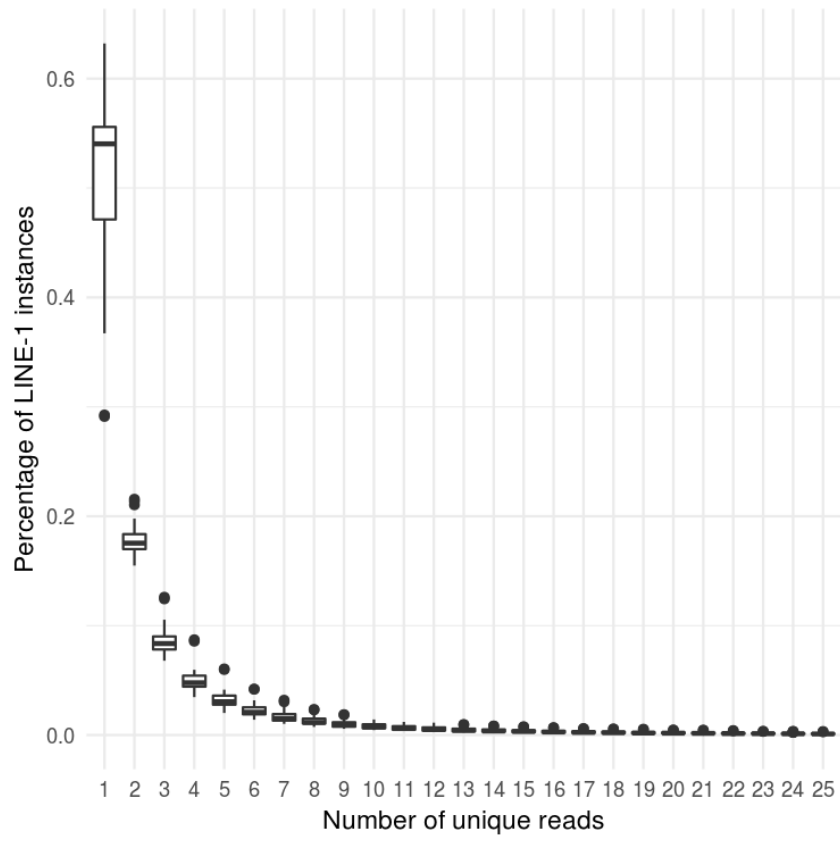

B.

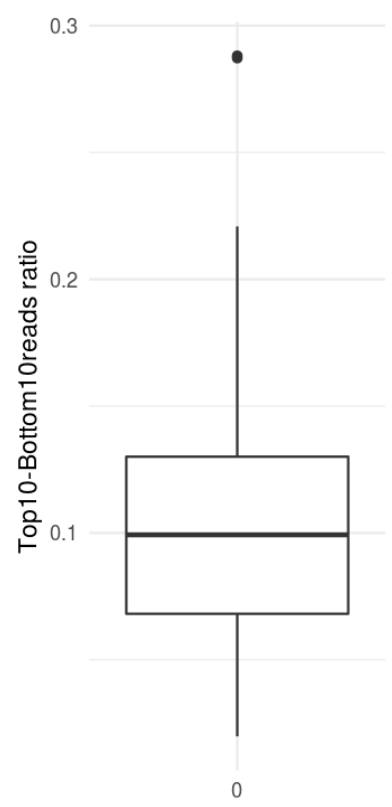

Supplement: S1 Fig — Broad model of pervasive transcription is evidenced by the number of uniquely mapped reads overlapping LINE-1 (L1Hs) instances. A. Most “expressed” LINE-1 (L1Hs) instances have small amount of uniquely mapped reads (1–5) suggesting low levels of transcription throughout the genome. B. Distribution of the read count ratio. Ratio between the Top10 mostly expressed instances and all instances having 10 reads or less across all GTEx RNA-seq assays–On average only 10% of the reads derive from top expressed L1Hs instances. (PDF) [file pcbi.1007293.s001.pdf]

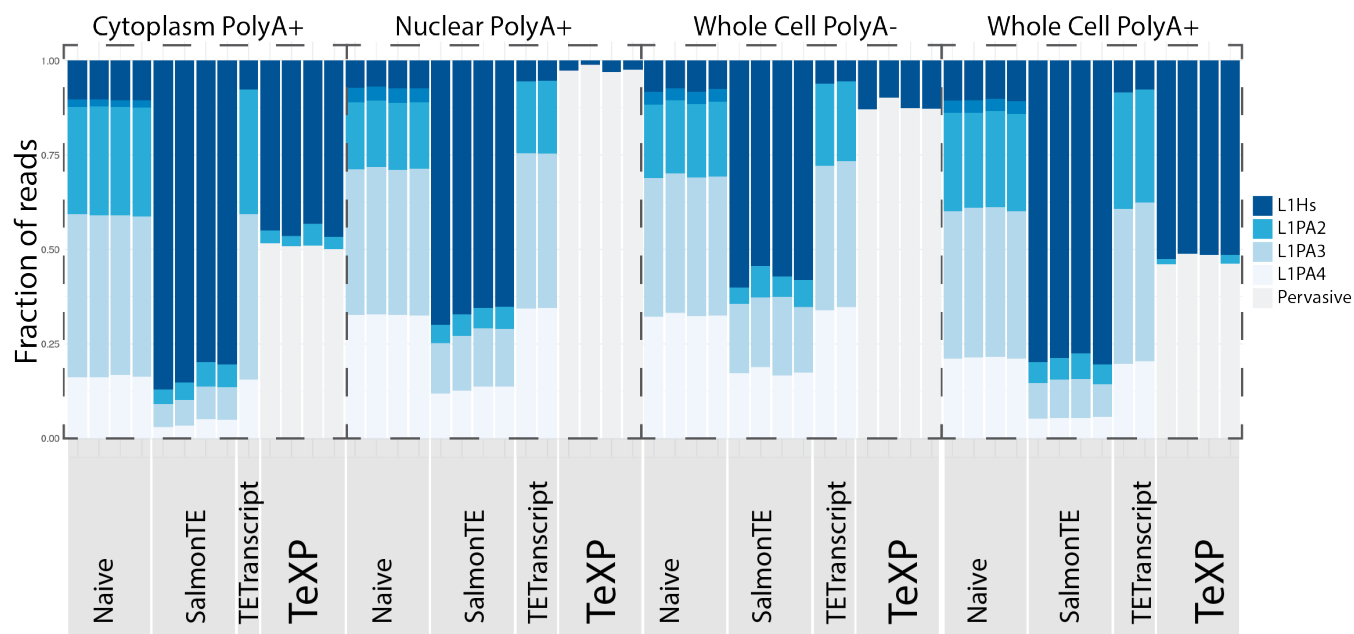

Supplement: S2 Fig — Fraction of reads deriving from 4 subfamilies and pervasive transcription from four methods. (PDF) [file pcbi.1007293.s002.pdf]

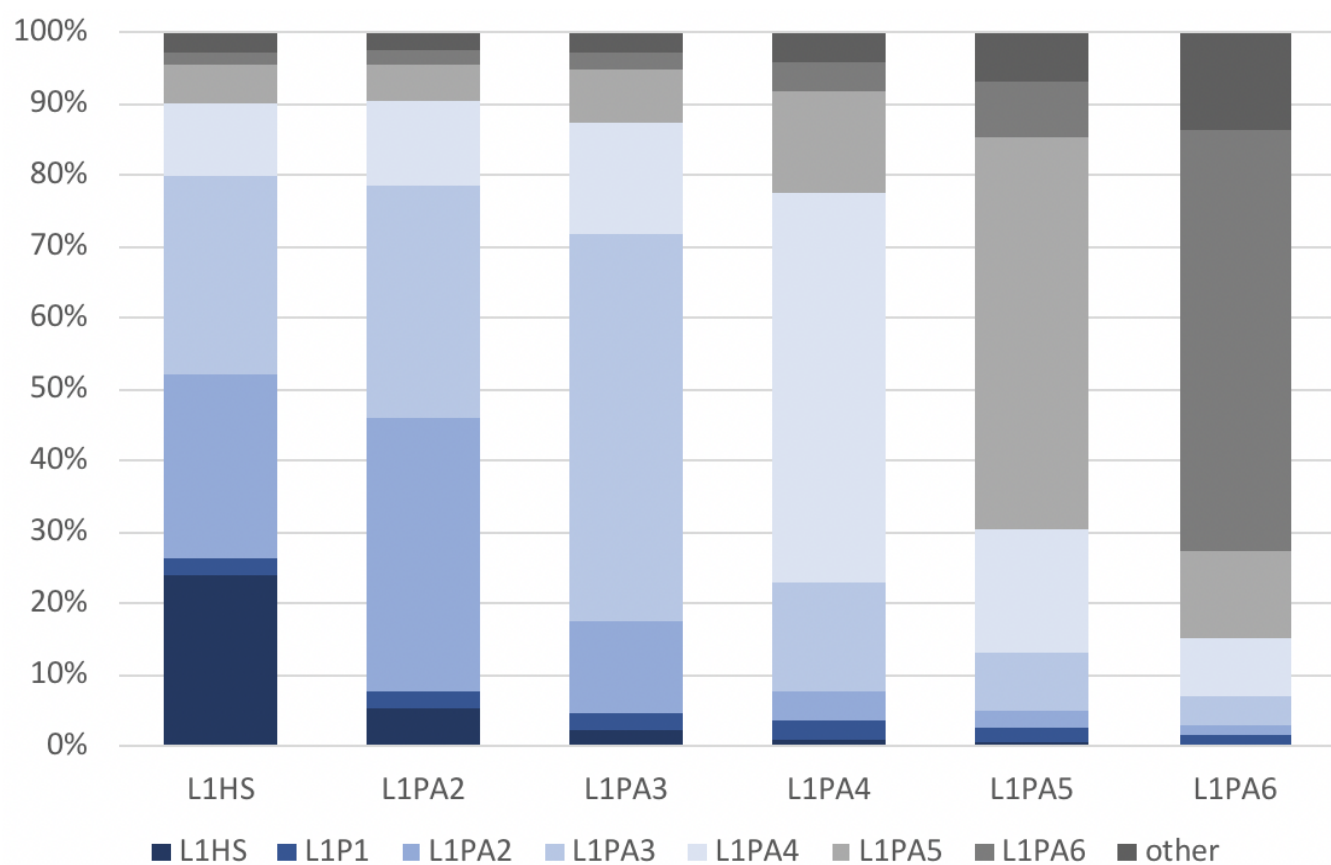

Supplement: S3 Fig — Simulated transcripts from putative L1Hs, L1P1, L1PA2, L1PA3, L1PA4, L1PA5 and L1PA6 were aligned to the reference genome using the same parameters as the TeXP pipeline. The proportion of reads mapped to each subfamily was calculated. (PDF) [file pcbi.1007293.s003.pdf]

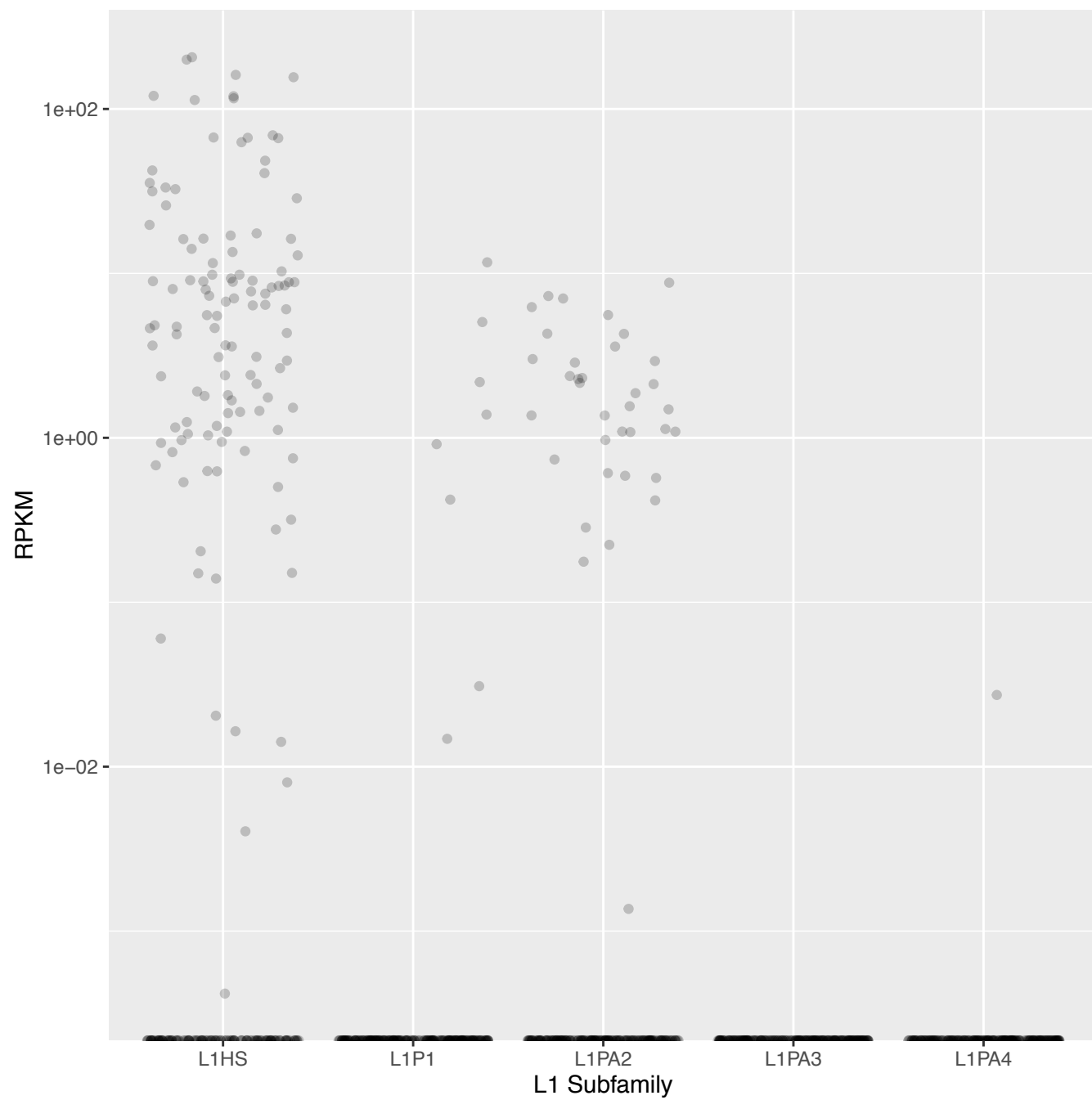

Supplement: S4 Fig — Most samples have zero RPKM (denser bar at the bottom). L1Hs has more samples with higher RPKM than any other subfamily followed by L1PA2 the second most recent L1 Subfamily. (PDF) [file pcbi.1007293.s004.pdf]

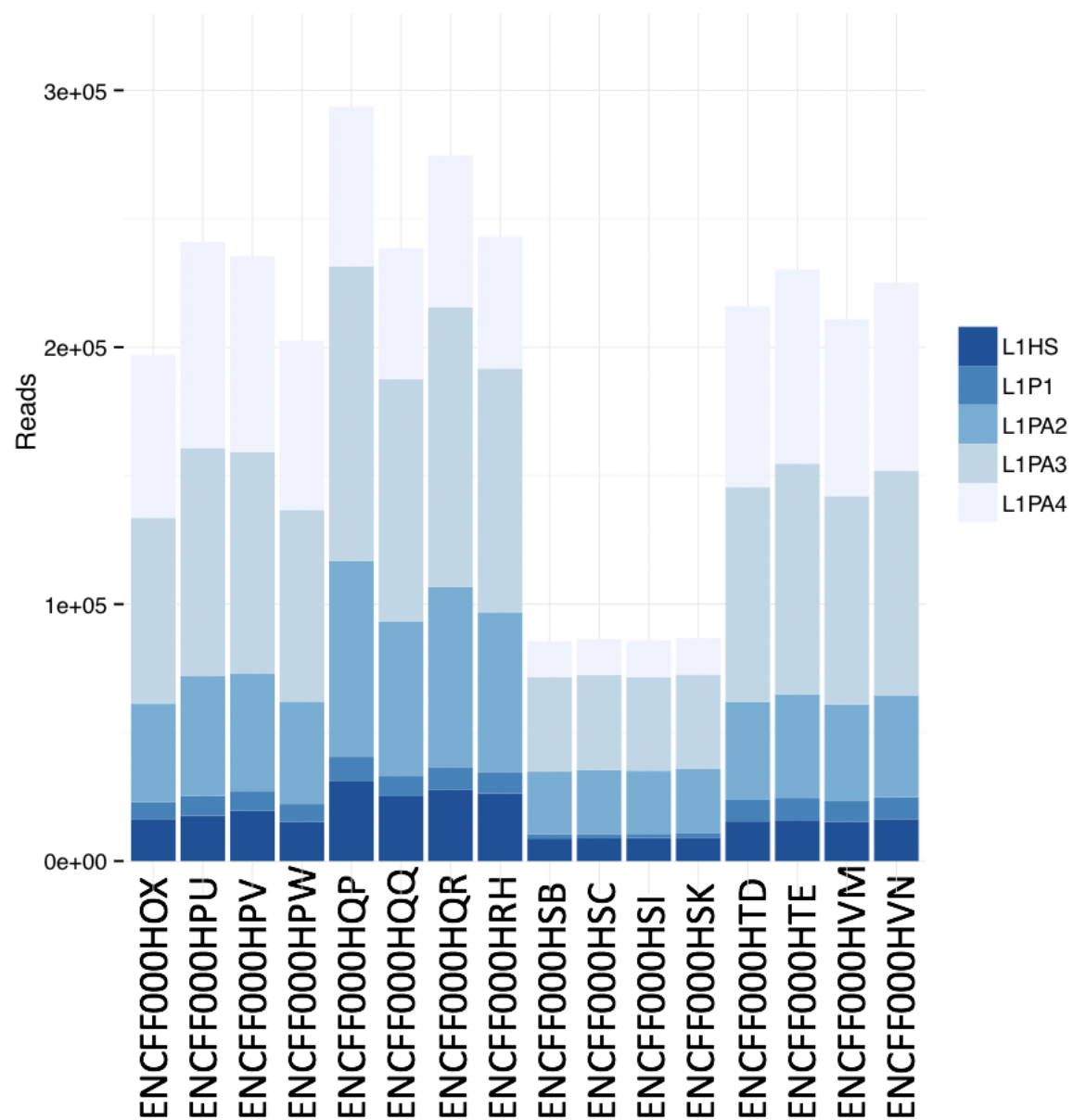

Supplement: S5 Fig — Every four bars are experiments respectively from whole cell polyA-; whole cell polyA+; cytoplasm polyA+ and nuclear polyA+. (PDF) [file pcbi.1007293.s005.pdf]

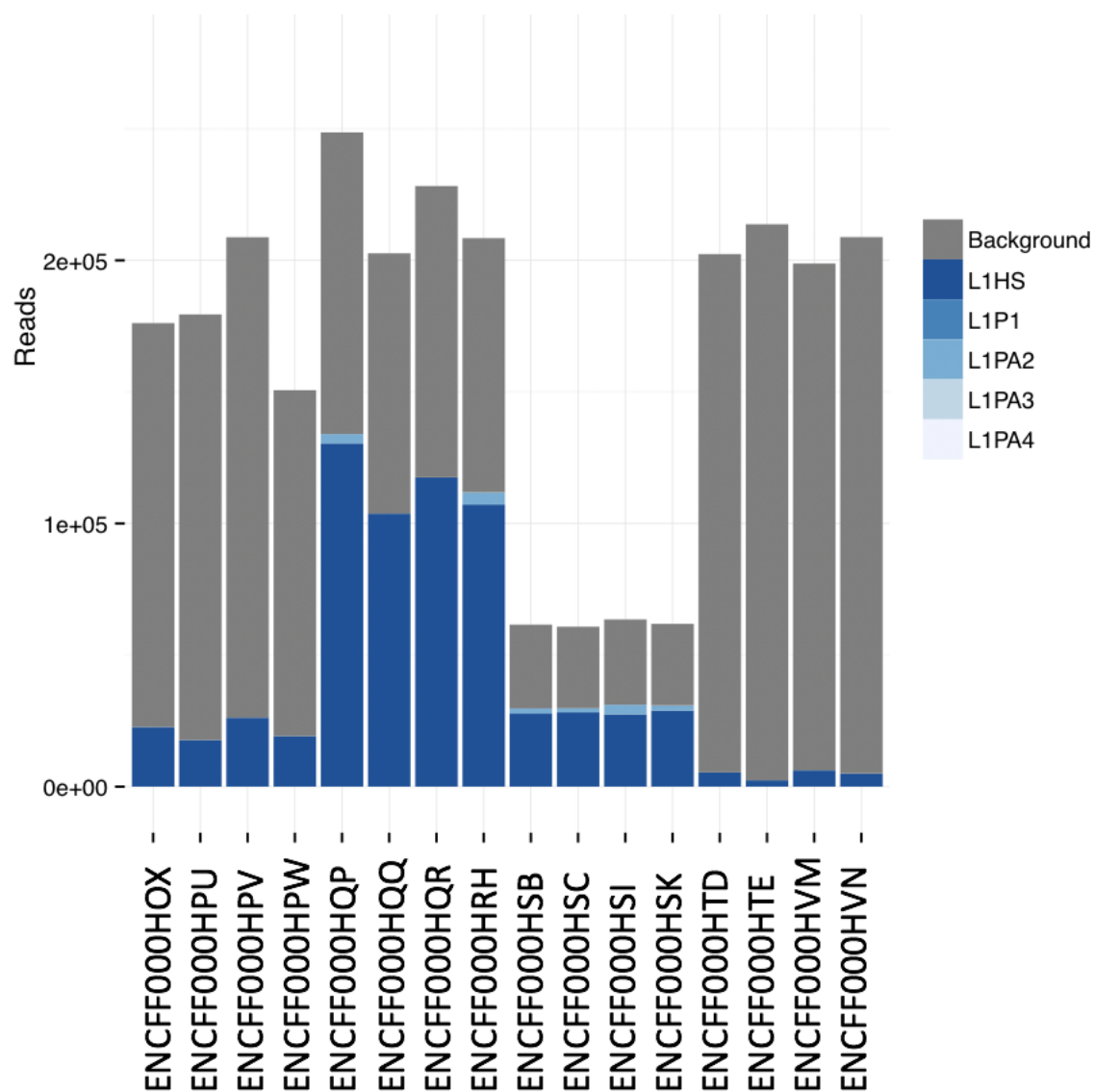

Supplement: S6 Fig — Every four bars are experiments respectively from whole cell polyA-; whole cell polyA+; cytoplasm polyA+ and nuclear polyA+. Gray and Dark blue bars refer to pervasive transcription and L1Hs autonomous transcription signal respectively. (PDF) [file pcbi.1007293.s006.pdf]

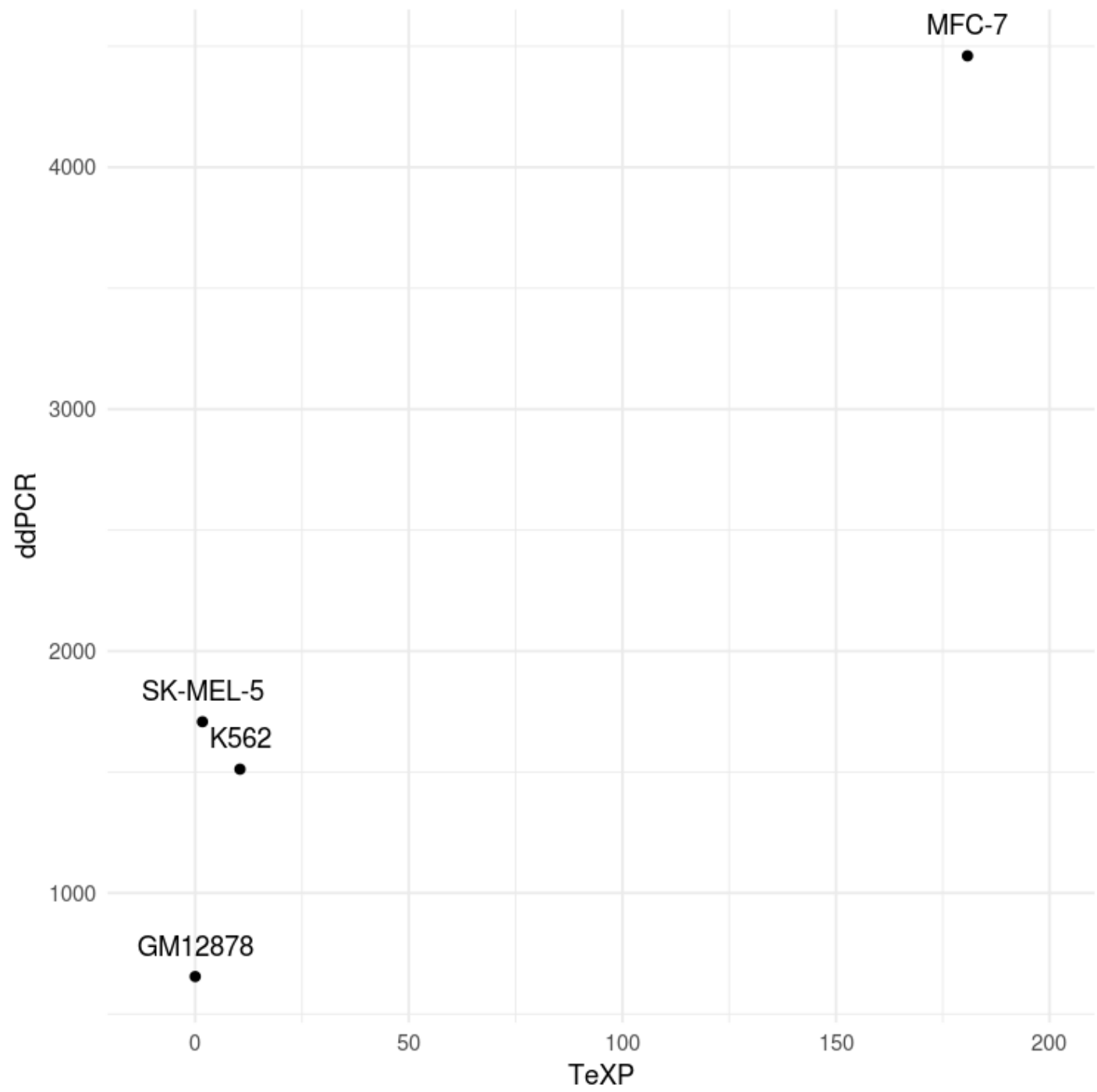

Supplement: S7 Fig — Estimates of L1Hs autonomous transcription from ddPCR (Y-axis) and TeXP (X-axis). (Spearman correlation, rho = 0.99, p-value = 3.803e-06) (PDF) [file pcbi.1007293.s007.pdf]

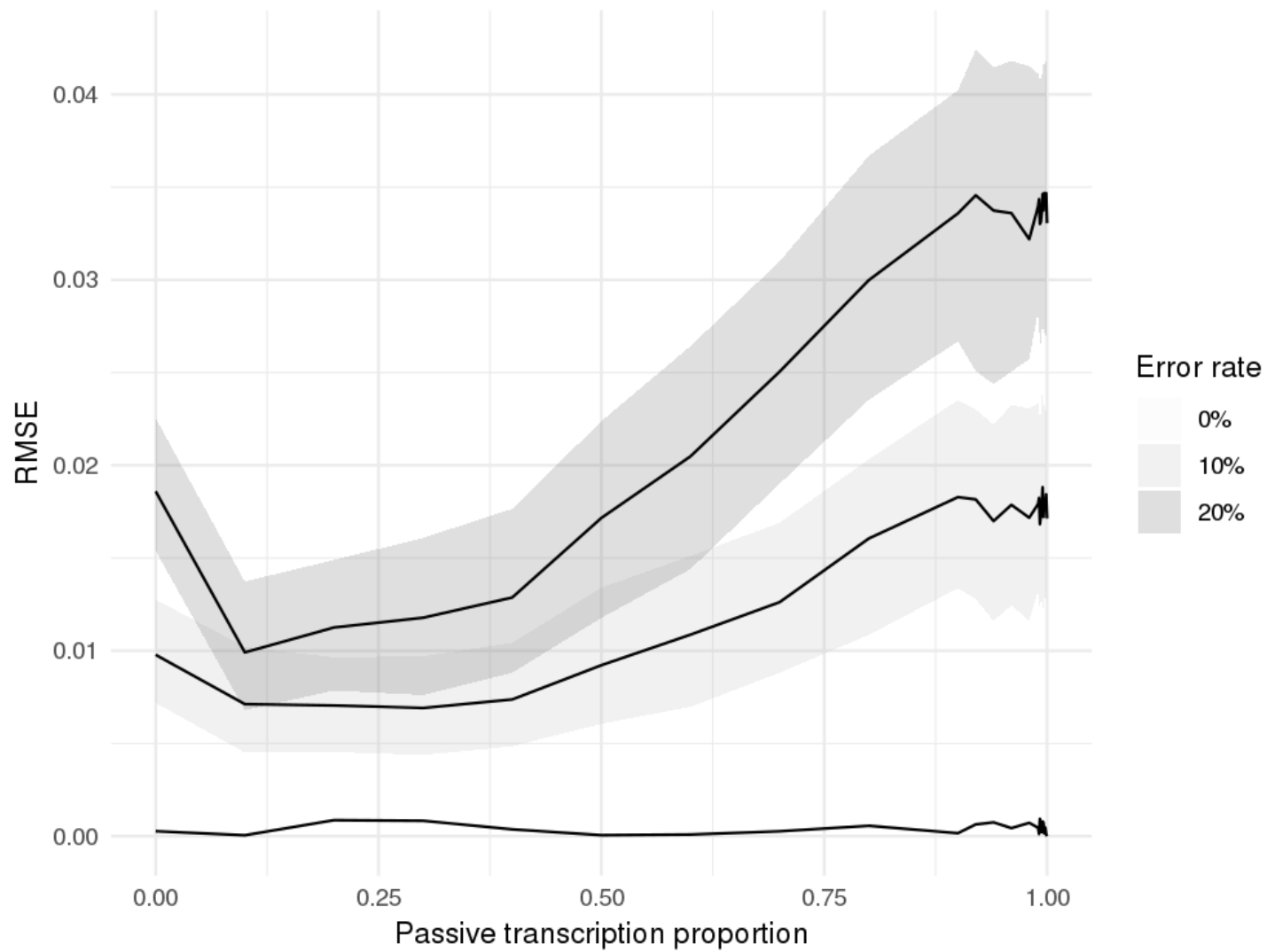

Supplement: S8 Fig — Input to TeXP were simulated as a combination of known signals (i.e. X pervasive transcription + (1-X) L1Hs autonomous transcription). Y-axis represents the root means square error for TeXP estimations at 3 different scenarios (0%, 10% and 20% error rate). Ribbons represent 25%-75% of TeXP rmse. (PDF) [file pcbi.1007293.s008.pdf]

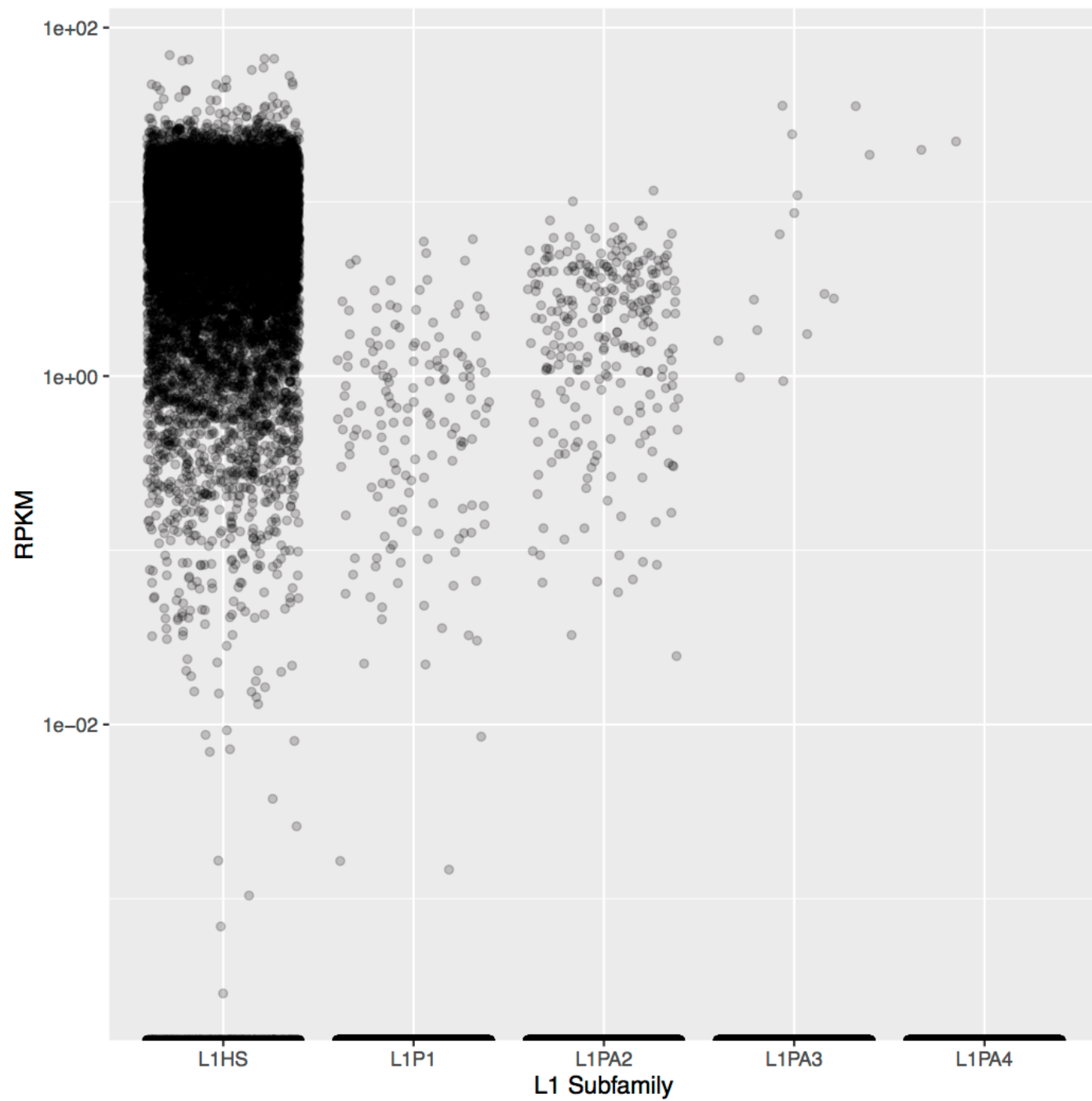

Supplement: S9 Fig — Most samples have zero RPKM (more dense bar at the bottom). L1Hs has more samples with higher RPKM than any other subfamily followed by L1PA2 the second most recent L1 Subfamily. (PDF) [file pcbi.1007293.s009.pdf]

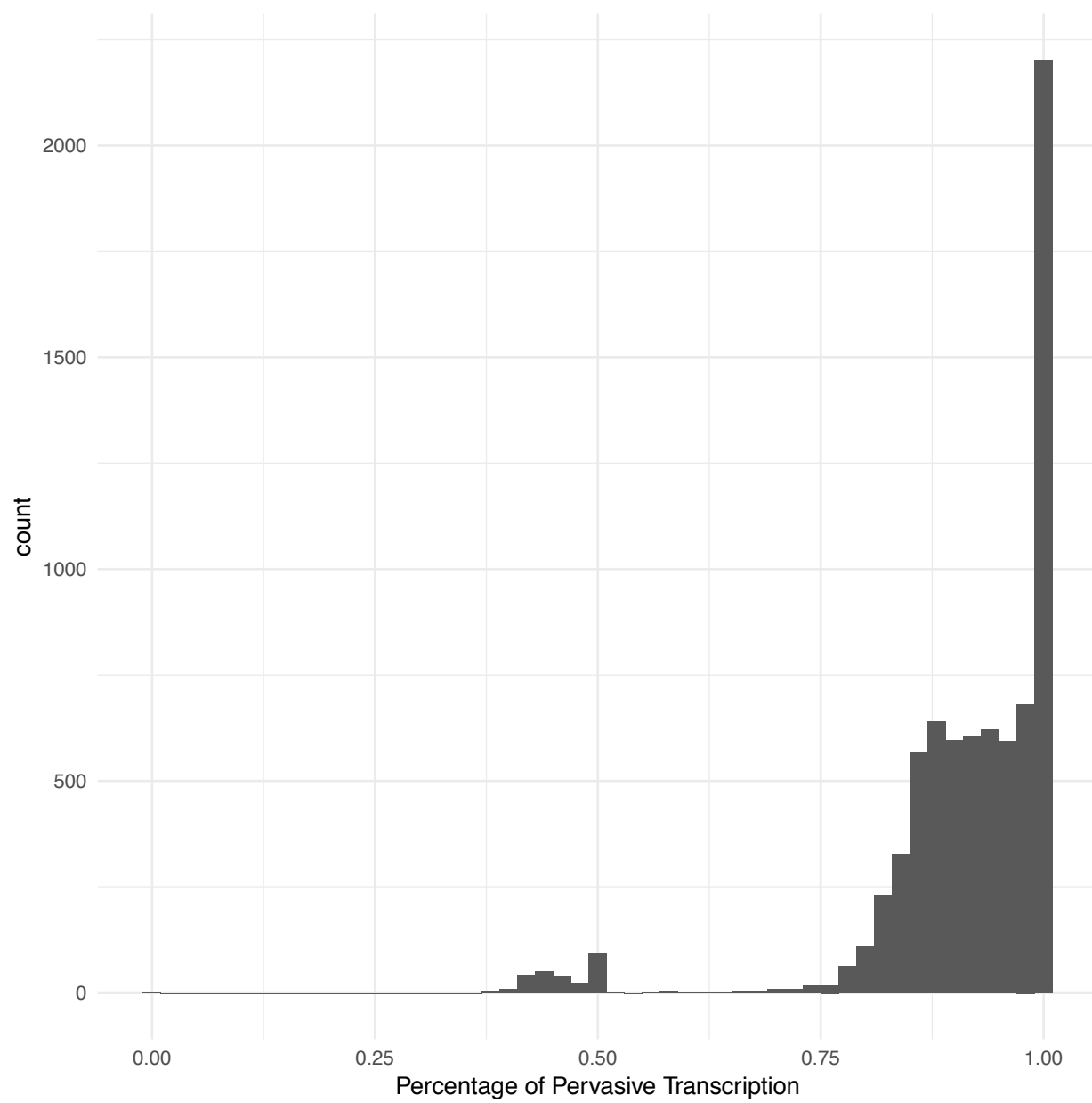

Supplement: S10 Fig — Most signal mapping to LINE-1 samples is derived from pervasive transcription. (PDF) [file pcbi.1007293.s010.pdf]

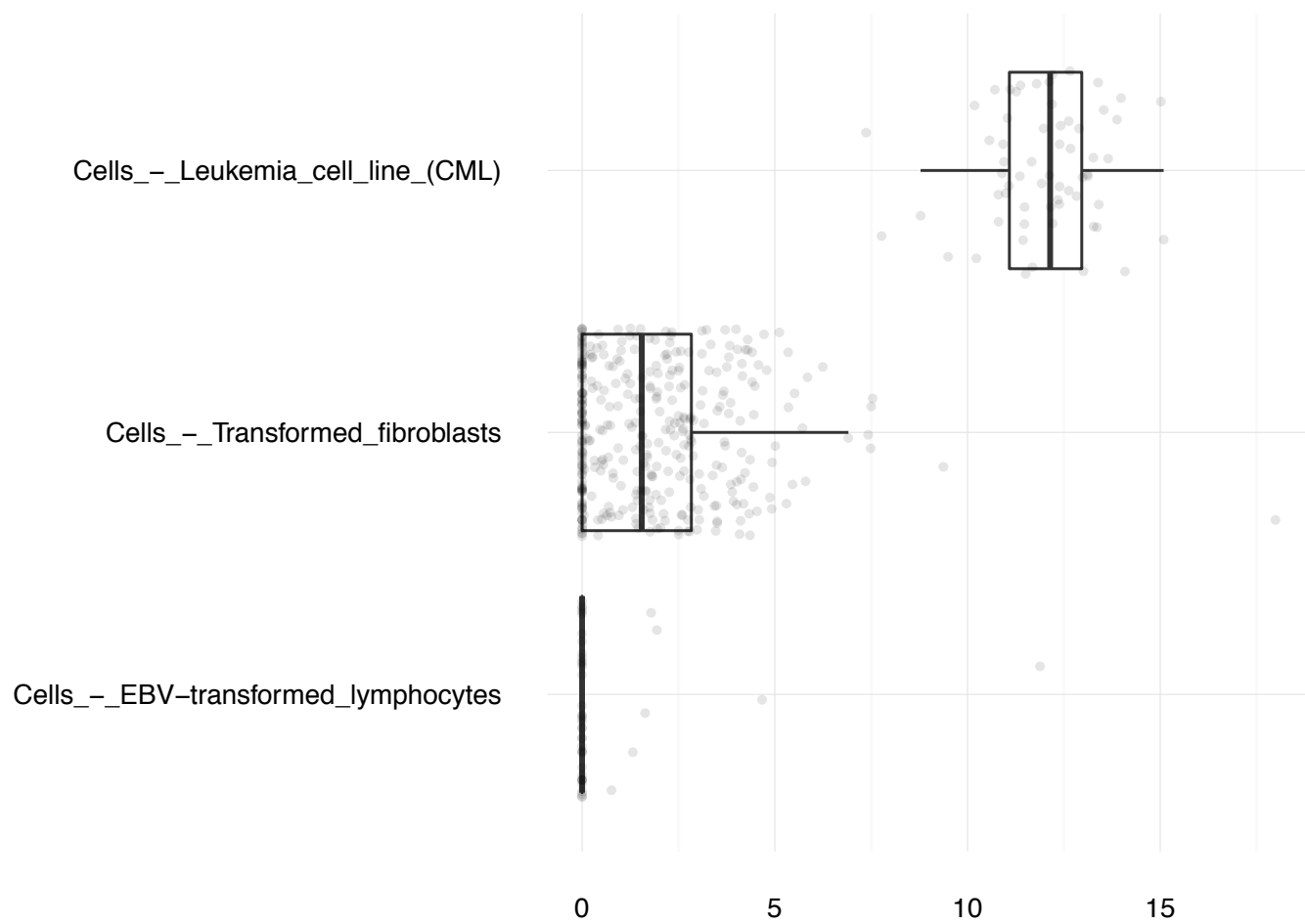

Supplement: S11 Fig — Most of EBV transformed cell lines have no autonomous transcription of L1Hs (bottom box). Transformed fibroblasts, derived from skin, have intermediate autonomous transcription of L1Hs (at lower levels than Skin samples). And K-562, derived from Leukemia tumor, has consistently high autonomous transcription of L1Hs across distinct batches. (PDF) [file pcbi.1007293.s011.pdf]

**A.**

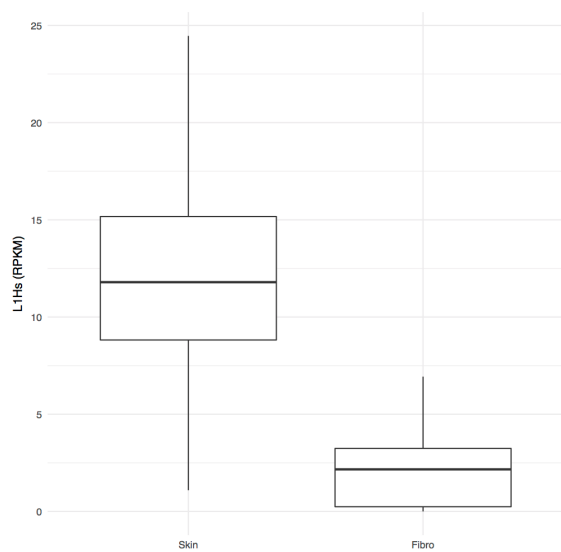

**B.**

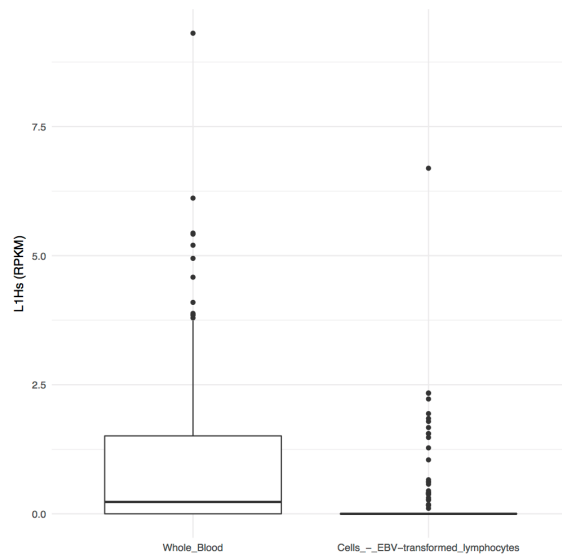

Supplement: S12 Fig — When comparing primary tissue and EBV transformed cell-lines from the same individuals we noticed a consistent decrease in the autonomous transcription of (A) Skin samples and EBV-Transformed fibroblasts (t = 22.5743, df = 153.878, p-value < 2.2e-16) and (B) Whole-blood and EBV-Transformed lymphocytes (t = 4.8937, df = 182.036, p-value = 2.171e-06). (PDF) [file pcbi.1007293.s012.pdf]

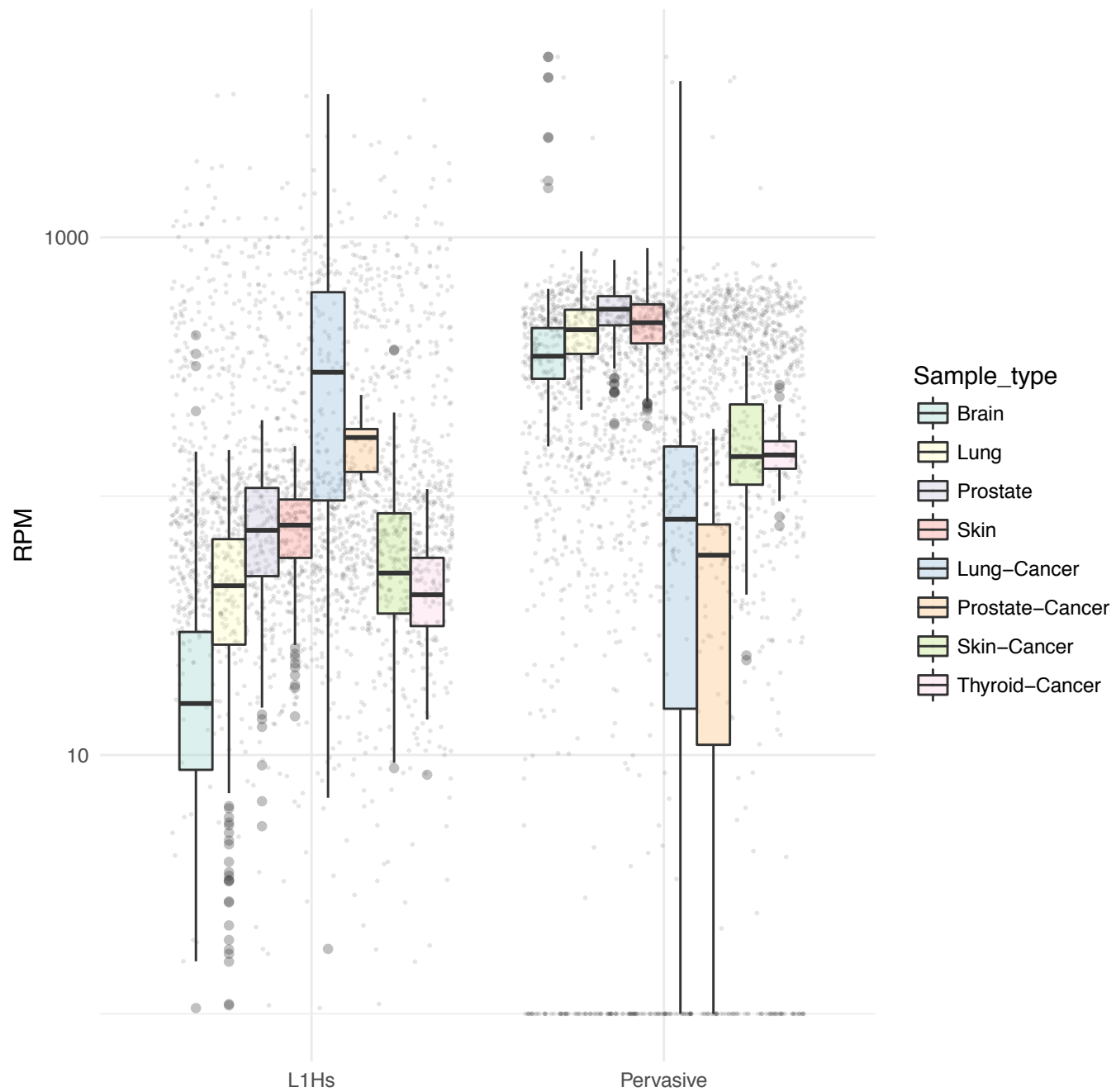

Supplement: S13 Fig — Distribution of normalized number of reads (RPM) derived from pervasive transcription and autonomous transcription of LINE-1 from tumor and healthy samples. (PDF) [file pcbi.1007293.s013.pdf]

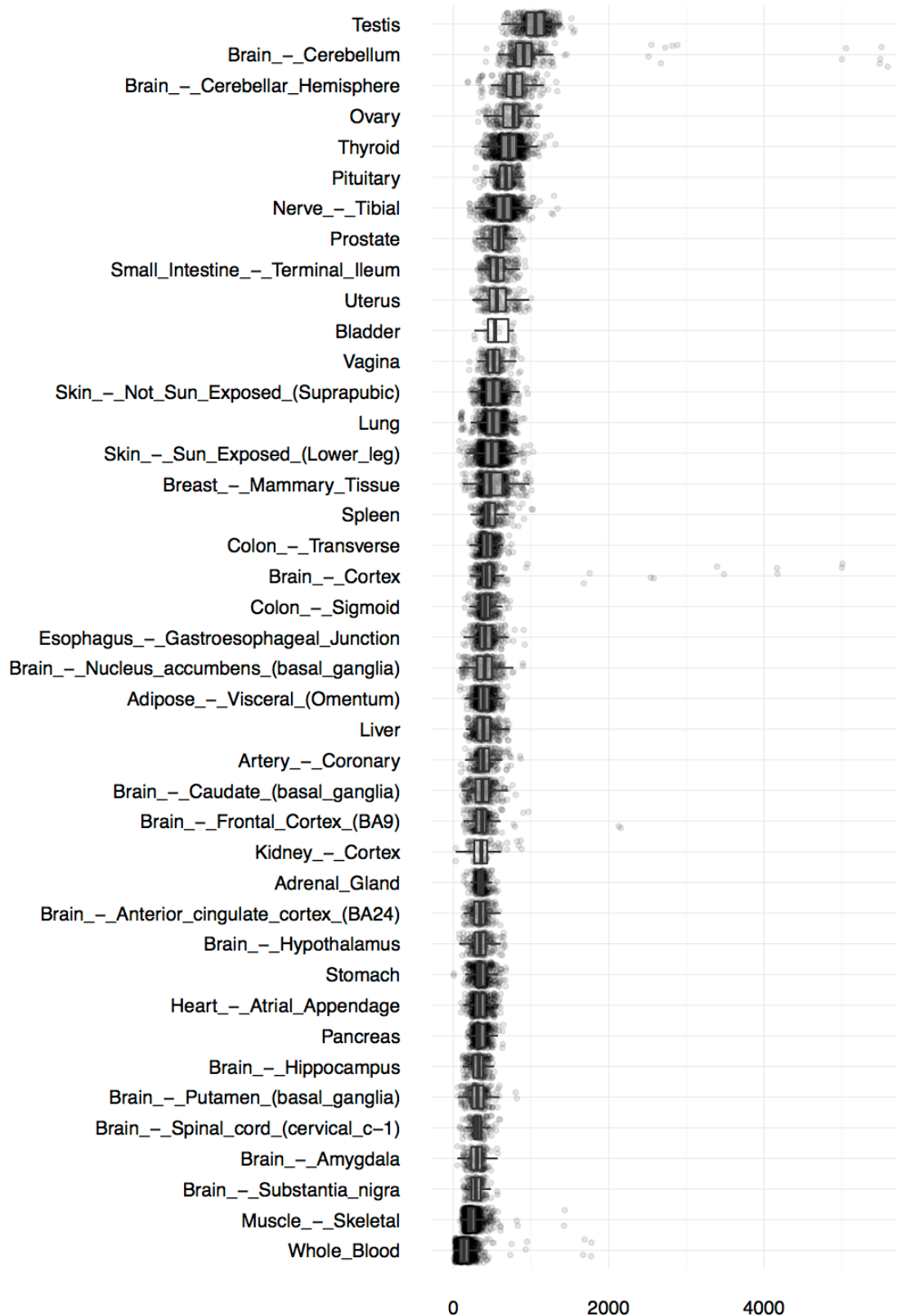

Supplement: S14 Fig — Pervasive transcription index was estimated for each GTEx sample and ordered by pervasive transcription median. (PDF) [file pcbi.1007293.s014.pdf]

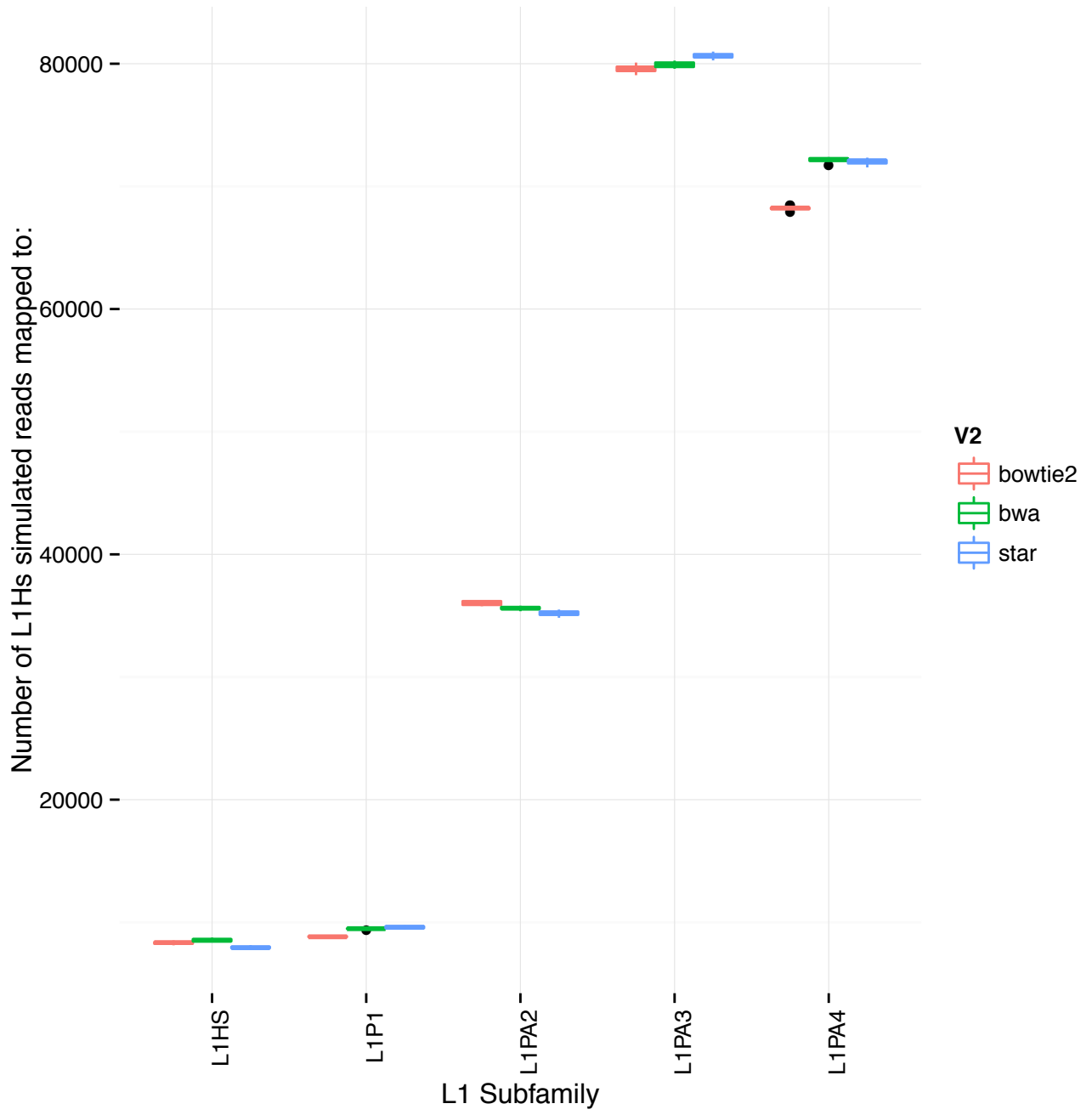

Supplement: S15 Fig — Different aligners were used to assess the construction of mappability fingerprints in the human reference genome. One hundred independent simulations of L1Hs transcript reads were independently mapped to the reference genome using bowtie2 (red), bwa (green) and star (blue). The box plot represents the distribution of the number of reads mapped to each L1 subfamily in the reference genome. (PDF) [file pcbi.1007293.s015.pdf]

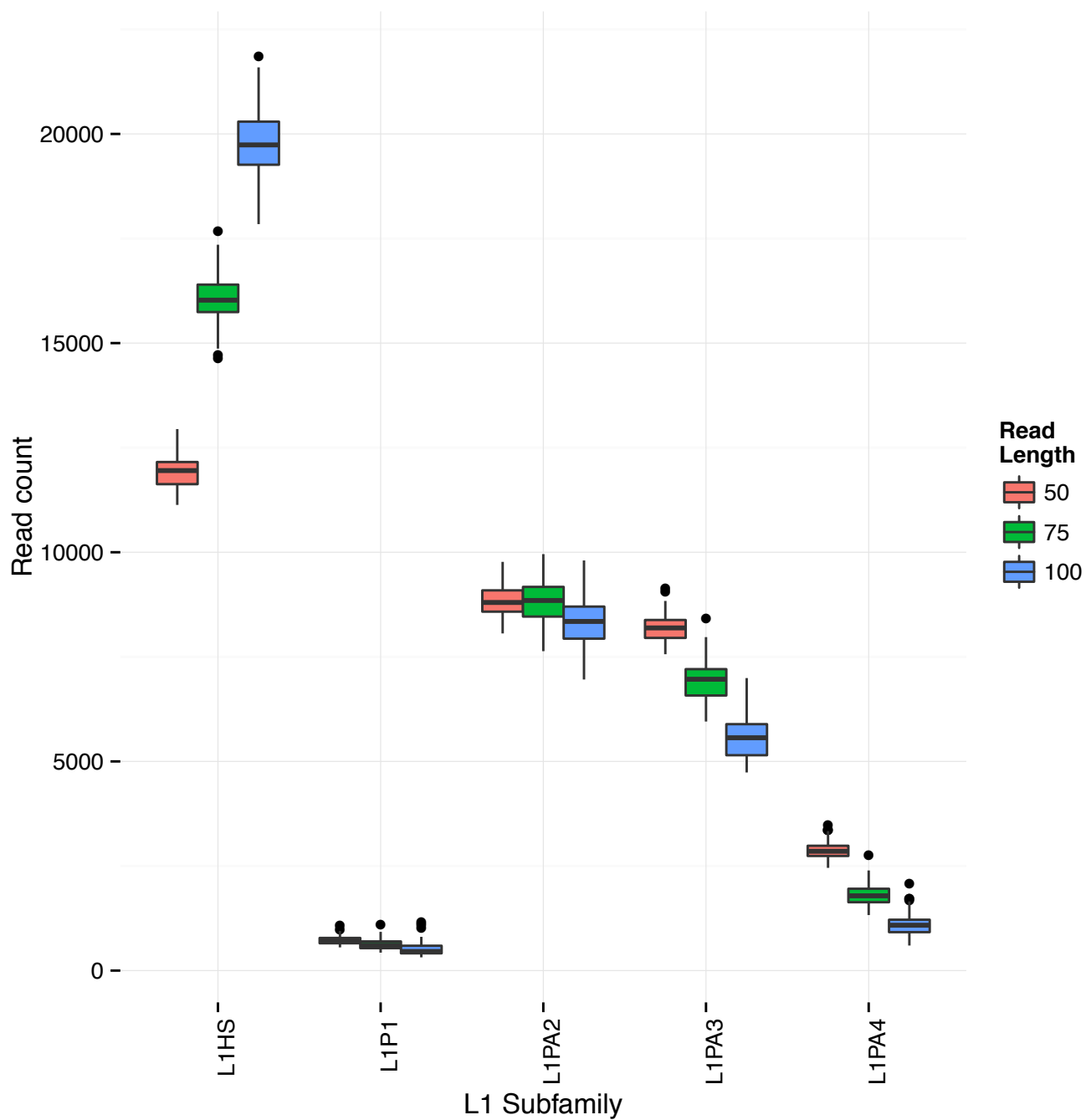

Supplement: S16 Fig — Simulating L1Hs reads with different length yield distinct proportions of reads mapped to each subfamily. As expected, the longer the read, the higher the proportion of reads correctly mapped originating subfamily. (PDF) [file pcbi.1007293.s016.pdf]

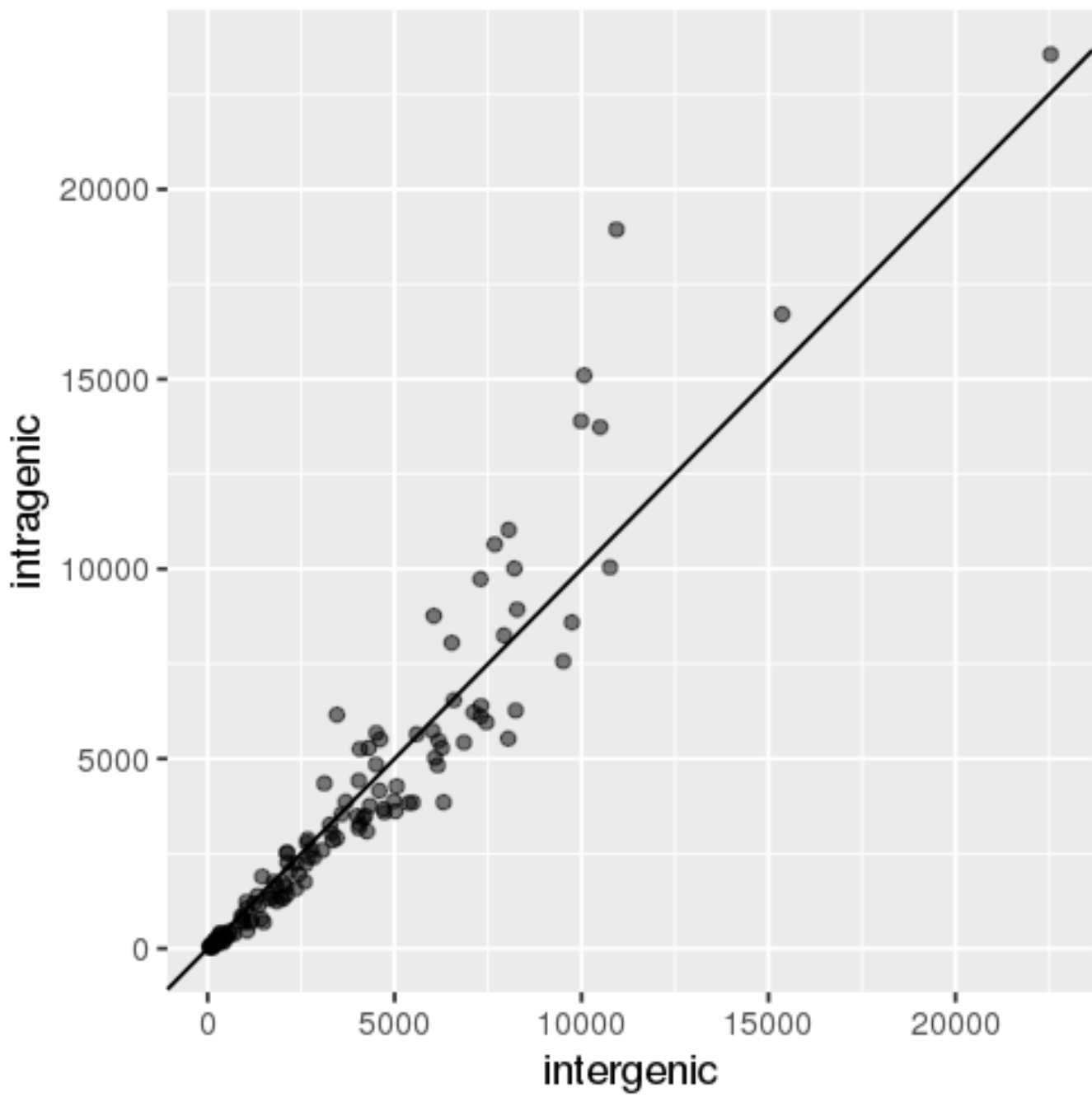

Supplement: S17 Fig — Correlation between the number of LINE-1 elements in intergenic (x-axis) and intronic (y-axis) regions (Spearman corr = 0.979057, p-value < 2.2e-16). (PDF) [file pcbi.1007293.s017.pdf]
