# Supplementary material for: TeXP: Deconvolving the effects of pervasive and autonomous transcription of transposable elements
Source: PLoS Comput Biol. 2019 Aug 19;15(8):e1007293. doi: 10.1371/journal.pcbi.1007293 (PMC6715295; doi:10.1371/journal.pcbi.1007293)
Supplement: S1 Table — (PDF) [file pcbi.1007293.s018.pdf]

ENCODE ID	Cell-line	Fraction	rRNA treatment	Transcript selection	L1Hs RPKM
ENCSR000AED	GM12878	whole_cell	rRNA-depleted	Poly-A+	0.00
ENCSR000AEC	GM12878	whole_cell	rRNA-depleted	Total	0.00
ENCSR000CQE	GM12878	cytoplasmic	rRNA-depleted	DSN_normalized_Poly-A-	0.00
ENCSR000COR	GM12878	cytoplasmic	rRNA-depleted	Poly-A+	0.00
ENCSR000CQF	GM12878	nuclear	rRNA-depleted	DSN_normalized_Poly-A-	0.00
ENCSR000CPO	GM12878	nuclear	rRNA-depleted	Poly-A+	0.00
ENCSR000CVT	GM12878	nucleolar	rRNA-depleted	Total	0.00
ENCSR000COS	GM12878	whole_cell	rRNA-depleted	DSN_normalized_Poly-A-	0.00
ENCSR000COQ	GM12878	whole_cell	rRNA-depleted	Poly-A+	0.00
ENCSR000CQT	HeLa-S3	cytoplasmic	rRNA-depleted	DSN_normalized_Poly-A-	0.00
ENCSR000CPP	HeLa-S3	cytoplasmic	rRNA-depleted	Poly-A+	4.09
ENCSR000CQI	HeLa-S3	nuclear	rRNA-depleted	DSN_normalized_Poly-A-	0.00
ENCSR000CPQ	HeLa-S3	nuclear	rRNA-depleted	Poly-A+	0.07
ENCSR000CQJ	HeLa-S3	whole_cell	rRNA-depleted	DSN_normalized_Poly-A-	0.00
ENCSR000CPR	HeLa-S3	whole_cell	rRNA-depleted	Poly-A+	0.00
ENCSR000CQU	HepG2	cytoplasmic	rRNA-depleted	DSN_normalized_Poly-A-	0.04
ENCSR000CPF	HepG2	cytoplasmic	rRNA-depleted	Poly-A+	1.70
ENCSR000CQK	HepG2	nuclear	rRNA-depleted	DSN_normalized_Poly-A-	0.00
ENCSR000CPC	HepG2	nuclear	rRNA-depleted	Poly-A+	0.00
ENCSR000CPD	HepG2	whole_cell	rRNA-depleted	DSN_normalized_Poly-A-	0.00
ENCSR000CPE	HepG2	whole_cell	rRNA-depleted	Poly-A+	0.00
ENCSR166QLP	HT1080	cytoplasmic	rRNA-depleted	Poly-A+	0.00
ENCSR535VTR	HT1080	whole_cell	rRNA-depleted	Total	0.68
ENCSR067UNX	HT1080	nuclear	rRNA-depleted	Poly-A+	0.00
ENCSR000AEM	K562	whole_cell	rRNA-depleted	Poly-A+	10.48
ENCSR000AEL	K562	whole_cell	rRNA-depleted	Total	8.40
ENCSR000CPY	K562	chromatin	rRNA-depleted	Total	2.78
ENCSR000CQL	K562	cytoplasmic	rRNA-depleted	DSN_normalized_Poly-A-	0.66
ENCSR000COK	K562	cytoplasmic	rRNA-depleted	Poly-A+	7.12
ENCSR000CQM	K562	nuclear	rRNA-depleted	DSN_normalized_Poly-A-	13.64

ENCSR000CPS	K562	nuclear	rRNA-depleted	Poly-A+	1.16
ENCSR000CPZ	K562	nucleolar	rRNA-depleted	Total	0.00
ENCSR000CQA	K562	nucleoplasm	rRNA-depleted	Total	0.00
ENCSR000CPG	K562	whole_cell	rRNA-depleted	DSN_normalized_Poly-A-	0.00
ENCSR000CPH	K562	whole_cell	rRNA-depleted	Poly-A+	2.29
ENCSR000CTU	MCF-7	cytoplasmic	rRNA-depleted	Poly-A+	33.22
ENCSR000CTO	MCF-7	nuclear	rRNA-depleted	Poly-A+	6.21
ENCSR000CQB	MCF-7	whole_cell	rRNA-depleted	DSN_normalized_Poly-A-	48.56
ENCSR000CPT	MCF-7	whole_cell	rRNA-depleted	Poly-A+	180.78
ENCSR586SEE	NCI-H460	cytoplasmic	rRNA-depleted	Poly-A+	14.83
ENCSR164OCT	NCI-H460	whole_cell	rRNA-depleted	Total	11.52
ENCSR625QJI	NCI-H460	nuclear	rRNA-depleted	Poly-A+	1.12
ENCSR291DJH	SK-MEL-5	cytoplasmic	rRNA-depleted	Poly-A+	17.63
ENCSR669KQU	SK-MEL-5	whole_cell	rRNA-depleted	Total	8.78
ENCSR201WVA	SK-MEL-5	nuclear	rRNA-depleted	Poly-A+	6.71
ENCSR569JKX	SK-N-DZ	cytoplasmic	rRNA-depleted	Poly-A+	1.16
ENCSR136WGP	SK-N-DZ	whole_cell	rRNA-depleted	Total	1.88
ENCSR255NYQ	SK-N-DZ	nuclear	rRNA-depleted	Poly-A+	0.61
ENCSR000CTR	SK-N-SH	cytoplasmic	rRNA-depleted	Poly-A+	0.69
ENCSR000CTS	SK-N-SH	nuclear	rRNA-depleted	Poly-A+	0.04
ENCSR000CQP	SK-N-SH	whole_cell	rRNA-depleted	DSN_normalized_Poly-A-	0.00
ENCSR000CPN	SK-N-SH	whole_cell	rRNA-depleted	Poly-A+	5.61
ENCSR000CTT	SK-N-SH	whole_cell	rRNA-depleted	Poly-A+	0.00
