# Supplementary material for: TeXP: Deconvolving the effects of pervasive and autonomous transcription of transposable elements
Source: PLoS Comput Biol. 2019 Aug 19;15(8):e1007293. doi: 10.1371/journal.pcbi.1007293 (PMC6715295; doi:10.1371/journal.pcbi.1007293)
Supplement: S2 Table — (PDF) [file pcbi.1007293.s019.pdf]

	Assay Name	Sequence (5’ → 3’)
FAM Labeled	L1Hs ORF1 FWD	ACAAAGCTGGATGGAGAATG
	L1Hs ORF1 REV	GTTTGAATGTCCTCCCGTAG
	L1Hs ORF1 Probe	ACGAGCTGAGAGAAGAAGGCT
	L1Hs ORF2 FWD	AAATACCATTTGACCCAGCC
	L1Hs ORF2 REV	ATACGTGTGCATGTGTCTTT
	L1Hs ORF2 Probe	TCCCATTACTGGGTATATACCCA
HEX Labeled	*HPRT1* 5’ End FWD	ACCAGGTTATGACCTTGATTT
	HPRT1 5’ End REV	TCCATGAGGAATAAACACCC
	HPRT1 5’ End Probe	TGCATACCTAATCATTATGCTGAGGA
	HPRT1 3’ End FWD	CCAGACAAGTTTGTTGTAGGA
	HPRT1 3’ End REV	CCAGTTTCACTAATGACACAAA
	HPRT1 3’ End Probe	CCCTTGACTATAATGAATACTTCAGGG
