# Supplementary material for: TeXP: Deconvolving the effects of pervasive and autonomous transcription of transposable elements
Source: PLoS Comput Biol. 2019 Aug 19;15(8):e1007293. doi: 10.1371/journal.pcbi.1007293 (PMC6715295; doi:10.1371/journal.pcbi.1007293)
Supplement: S3 Table — Comparison of the absolute expression of copies of full-length transcript/ng of L1Hs autonomous transcript (ORF1) and L1Hs pervasive transcript (ORF2) when run with both references. (PDF) [file pcbi.1007293.s020.pdf]

	Reference	**MFC-7**	**K562**	**SK-MEL-5**	**GM12878**	**HeLa**	**HepG2**
**ORF1-Autonomous Transcription** (copies of full-length transcript/ng)	*HPRT1* 5' End	4460	1512	1708	655	696	964
	HPRT1 3' End	3370	1604	1810	735	709	1028
**ORF2-Pervasive Transcription** (copies of truncated transcript/ng)	*HPRT1* 5' End	12600	2838	3562	2855	4004	3916
	HPRT1 3' End	14050	3136	3720	2975	4381	4482
ddPCR estimation of autonomous transcription	--	2,412.8	990.0	1,075.6	233.0	132.0	378.4
TeXP Estimation of autonomous transcription (Whole-cell PolyA+)	--	180.78	8.40	8.78	0	0	1.70
