# Supplementary material for: TeXP: Deconvolving the effects of pervasive and autonomous transcription of transposable elements
Source: PLoS Comput Biol. 2019 Aug 19;15(8):e1007293. doi: 10.1371/journal.pcbi.1007293 (PMC6715295; doi:10.1371/journal.pcbi.1007293)
Supplement: S4 Table — Bladder, Kidney Cortex and minor salivary gland were eliminated from further analysis. (PDF) [file pcbi.1007293.s021.pdf]

| Number of Samples | Tissue |
|---|---|
| 234 | Adipose_-_Visceral_(Omentum) |
| 146 | Adrenal_Gland |
| 123 | Artery_-_Coronary |
| 11 | Bladder |
| 81 | Brain_-_Amygdala |
| 99 | Brain_-_Anterior_cingulate_cortex_(BA24) |
| 133 | Brain_-_Caudate_(basal_ganglia) |
| 115 | Brain_-_Cerebellar_Hemisphere |
| 145 | Brain_-_Cerebellum |
| 132 | Brain_-_Cortex |
| 117 | Brain_-_Frontal_Cortex_(BA9) |
| 102 | Brain_-_Hippocampus |
| 103 | Brain_-_Hypothalamus |
| 123 | Brain_-_Nucleus_accumbens_(basal_ganglia) |
| 103 | Brain_-_Putamen_(basal_ganglia) |
| 76 | Brain_-_Spinal_cord_(cervical_c-1) |
| 71 | Brain_-_Substantia_nigra |
| 200 | Breast_-_Mammary_Tissue |
| 132 | Cells_-_EBV-transformed_lymphocytes |
| 78 | Cells_-_Leukemia_cell_line_(CML) |
| 300 | Cells_-_Transformed_fibroblasts |
| 142 | Colon_-_Sigmoid |
| 178 | Colon_-_Transverse |
| 151 | Esophagus_-_Gastroesophageal_Junction |
| 192 | Heart_-_Atrial_Appendage |
| 36 | Kidney_-_Cortex |
| 136 | Liver |
| 280 | Lung |
| 69 | Minor_Salivary_Gland |
| 468 | Muscle_-_Skeletal |
| 335 | Nerve_-_Tibial |
| 108 | Ovary |
| 193 | Pancreas |
| 124 | Pituitary |
| 119 | Prostate |
| 271 | Skin_-_Not_Sun_Exposed_(Suprapubic) |

| | |
|---|---|
| 395 | Skin_-_Sun_Exposed_(Lower_leg) |
| 104 | Small_Intestine_-_Terminal_Ileum |
| 118 | Spleen |
| 205 | Stomach |
| 199 | Testis |
| 355 | Thyroid |
| 90 | Uterus |
| 88 | Vagina |
| 449 | Whole_Blood |
