# Supplementary material for: TeXP: Deconvolving the effects of pervasive and autonomous transcription of transposable elements
Source: PLoS Comput Biol. 2019 Aug 19;15(8):e1007293. doi: 10.1371/journal.pcbi.1007293 (PMC6715295; doi:10.1371/journal.pcbi.1007293)
Supplement: S5 Table — (PDF) [file pcbi.1007293.s022.pdf]

Tissue	correlation	FDR
Lung	0.2818789	7.22E-05
Muscle_-_Skeletal	0.1788474	2.20E-03
Cells_-_Transformed_fibroblasts	0.2089546	3.60E-03
Prostate	-0.3238307	3.60E-03
Adipose_-_Visceral_(Omentum)	0.2277305	3.93E-03
Skin_-_Not_Sun_Exposed_(Suprapubic)	0.1753624	2.77E-02
Whole_Blood	-0.1302921	3.58E-02
Breast_-_Mammary_Tissue	0.1858914	4.11E-02
Testis	0.1886884	4.11E-02
Skin_-_Sun_Exposed_(Lower_leg)	0.1147755	9.91E-02
Esophagus_-_Gastroesophageal_Junction	0.1791177	1.11E-01
Brain_-_Cerebellar_Hemisphere	0.1997148	1.19E-01
Brain_-_Frontal_Cortex_(BA9)	0.1880845	1.33E-01
Pituitary	0.182976	1.33E-01
Brain_-_Cortex	0.1588801	2.02E-01
Brain_-_Substantia_nigra	0.2120247	2.09E-01
Brain_-_Hippocampus	0.1600052	2.69E-01
Kidney_-_Cortex	0.2710293	2.69E-01
Heart_-_Atrial_Appendage	0.1102971	2.88E-01
Minor_Salivary_Gland	-0.1837433	2.88E-01
Uterus	0.139628	3.97E-01
Adrenal_Gland	0.102378	4.36E-01
Nerve_-_Tibial	0.06607081	4.36E-01
Brain_-_Anterior_cingulate_cortex_(BA24)	0.1186109	4.44E-01
Cells_-_EBV-transformed_lymphocytes	0.09962761	4.50E-01
Brain_-_Nucleus_accumbens_(basal_ganglia)	0.08814543	5.62E-01
Brain_-_Caudate_(basal_ganglia)	0.07846093	6.02E-01
Brain_-_Putamen_(basal_ganglia)	0.08561444	6.13E-01
Ovary	0.07199146	6.96E-01
Stomach	0.05014164	6.97E-01
Bladder	-0.2305936	7.03E-01
Brain_-_Cerebellum	-0.05408436	7.13E-01
Liver	0.04890585	7.19E-01
Small_Intestine_-_Terminal_Ileum	0.05828941	7.19E-01
Thyroid	0.03256656	7.19E-01

Spleen	-0.03437764	8.70E-01
Pancreas	0.02279996	8.95E-01
Colon_-_Sigmoid	0.02209352	9.19E-01
Brain_-_Amygdala	0.02406885	9.36E-01
Brain_-_Hypothalamus	-0.01603804	9.36E-01
Brain_-_Spinal_cord_(cervical_c-1)	-0.02072991	9.36E-01
Artery_-_Coronary	-0.008006473	9.74E-01
Colon_-_Transverse	0.001340182	9.86E-01
Vagina	0.003462678	9.86E-01
